# Ethnic Differences in Atrial Fibrillation in the United Kingdom

**DOI:** 10.1016/j.jacadv.2024.101043

**Published:** 2024-07-03

**Authors:** Emilie K. Frimodt-Møller, Janet J. Tang, Tor Biering-Sørensen, Francesca N. Delling, Larry R. Jackson, Gregory M. Marcus

**Affiliations:** a Division of Cardiology, University of California-San Francisco, San Francisco, California, USA; b Department of Cardiology, Herlev and Gentofte Hospital, University of Copenhagen, Copenhagen, Denmark; c Department of Biomedical Sciences, Faculty of Health and Medical Sciences, University of Copenhagen, Copenhagen, Denmark; d Division of Cardiology, Duke Clinical Research Institute, Duke University School of Medicine, Durham, North Carolina, USA

**Keywords:** atrial fibrillation, ethnicity, stroke, systemic infarcts, UK Biobank

## Abstract

**Background:**

Within the United States, White individuals experience a higher risk of atrial fibrillation (AF) while exhibiting a lower AF-related stroke risk compared to other ethnic groups. It is possible that these observations stem from phenomena unique to the United States, such as differential health care access. The United Kingdom provides socialized medicine, which ostensibly promotes equitable health care access.

**Objectives:**

The purpose of the study was to examine whether ethnic differences in the risks of AF and AF-related stroke and systemic infarcts exist in the United Kingdom.

**Methods:**

We leveraged longitudinal data from the UK Biobank between January 1, 2006, and June 30, 2020. Ethnicity was categorized as White, Black, South Asian, Chinese, or multiracial. Incident AF, stroke, and systemic infarct were ascertained from in- and out-patient records.

**Results:**

A total of 458,785 participants (438,333 White, 7,244 Black, 9,143 South Asian, 1,376 Chinese, and 2,689 multiracial) were included. After excluding those with prevalent AF, 8,706 developed incident AF. Black individuals (HR: 0.60; 95% CI: 0.49-0.74; *P* < 0.001), South Asians (HR: 0.59; 95% CI: 0.49-0.72; *P* < 0.001), and Chinese (HR: 0.31; 95% CI: 0.12-0.83; *P* = 0.002) each exhibited substantially lower risks of AF compared to White individuals after multivariable adjustment. In AF participants, incident stroke and systemic infarct occurred in 1,550. No significant differences in the risk of AF-related stroke and systemic infarcts by ethnicity were detected, but small numbers of these events were present.

**Conclusions:**

In a European population with universal access to health care, White individuals consistently experienced the highest risk of AF, but there were no detectable differences in AF-related stroke or systemic infarcts.

Atrial fibrillation (AF) is the most common sustained arrythmia worldwide and is associated with increased morbidity, including stroke, heart failure, and overall mortality.^1^, ^2^, ^3^ Within the United States, White individuals experience a higher risk of AF compared to other racial and ethnic populations.^4^, ^5^, ^6^, ^7^, ^8^, ^9^ The mechanisms underlying these phenomena are largely unknown, but several theories have been proposed, including the influence of inherited genetic variants more common with European ancestry^10^^,^^11^ or disparities in health care access leading to enhanced AF detection in white individuals.^9^

AF is the most common cause of ischemic stroke, increasing the risk by 4- to 5-fold.^12^ Racial and ethnic differences in AF-related ischemic stroke have also been reported in the U.S. population, with evidence that American Indian and Alaska Native populations, Hispanics, and non-Hispanic black individuals each experienced a higher risk of AF-related stroke compared to white individuals.^13^^,^^14^

The observations of racial and ethnic differences in AF and AF-related stroke might stem from phenomena unique to the United States, such as differential access to health care, social determinants of health, suboptimal clinician-patient communication, a lack of trust among some groups related to U.S.-specific research history,^15^ or issues pertinent to unique environments. The observed differences may represent something more universal to different racial and ethnic groups that may prove informative and useful to all AF patients, regardless of race or ethnicity. The United Kingdom provides socialized medicine, which at least affords the opportunity for equal access to health care regardless of race or ethnicity. To our knowledge, this is the first study using longitudinal data from the UK Biobank to examine whether ethnic differences in AF and AF-related stroke and systemic embolism risk persist in a country outside the United States.

## Methods

The UK Biobank is a publicly available population-based prospective cohort study, approved by the National Information Governance Board for Health and Social Care and the National Health Service (NHS) North West Multicenter Research Ethics Committee. Details about the UK Biobank have been described elsewhere.^16^ Recruitment was conducted between 2006 and 2010 through postal invites of individuals aged between 40 and 69 years identified through NHS registers. All individuals living within 10 miles of one of the 22 UK Biobank assessment centers were invited to participate. Around 500,000 participants were included in the study cohort, of which 94.5% were of White ethnicity. This distribution exhibited a slightly higher proportion of White individuals compared to the national population of the same age range in the 2011 UK Census for England, Wales, and Scotland (91.3%).^17^

Extensive questionnaire data (completed as self-administered touchscreen and nurse-led questionnaire), physical measures, and biological samples were collected at recruitment, with ongoing enhanced data collection in large subsets of the cohort. The participants also provided consent for prospective data linkage to national electronic health-related data sets. All participants provided informed consent.^16^

### Study population

Participants with ethnicity defined as missing, “do not know” or “prefer not to answer” (n = 2,777), or “other ethnic group” (n = 4,558) were excluded.

### Ethnicity

Ethnicity was self-identified and categorized as White (including British, Irish, or “any other White background”), Black (including Caribbean, African, or “any other Black background”), South Asian (including Indian, Pakistani, Bangladeshi, or “any other Asian background”), Chinese, or multiracial (including White and Black Caribbean, White and Black African, White and Asian, or “any other mixed background”). Based on the geographic differentiation of South Asian and Chinese and collapsing of categories as described above, and in accordance with the UK NHS’s ethnicity categories,^18^ the groups were categorized as ethnicities rather than races.

### Covariate assessment

Baseline age and sex were obtained from the initial assessment visit. Educational level was self-reported. Body mass index was derived from height and weight measurements at the initial assessment and calculated as weight in kilograms divided by height in meters squared (kg/m^2^). Information on baseline comorbidities including diabetes, hypertension, coronary disease, valvular disease, congestive heart failure, chronic kidney disease, and obstructive sleep apnea were considered present if self-reported or if listed as primary or secondary diagnosis in in- and out-patient reports with International Classification of Diseases-9th Revision (ICD-9) and International Statistical Classification of Diseases and Related Health Problems-10th Revision (ICD-10) codes by the time of the first study visit (Supplemental Table 1). Smoking and alcohol use were self-reported at recruitment.

### Outcome ascertainment

Incident outcomes, including AF, ischemic stroke, and systemic infarcts were ascertained from January 1, 2006, through June 30, 2020, and defined as not having the condition prior to (and including) recruitment but subsequently developing the diagnosis during the study period. AF (including paroxysmal, persistent, and chronic AF), ischemic stroke (including nonhemorrhagic stroke and transient ischemic attack), and systemic infarctions (including retinal infarctions, ischemic deafness, pulmonary embolisms and infarctions, aorta, mesenteric, hepatic, and renal infarctions, infarctions in the extremities, and embolism and thrombosis of other specified artery) were assessed from in- and out-patient reports determined by ICD-9 and ICD-10 codes for the diseases listed as either a primary or secondary diagnosis (Supplemental Table 1). Myocardial infarction was not included since the etiology of myocardial infarction may more likely be due to coronary atherosclerosis than embolism.

### Statistical analysis

Normally distributed continuous variables are described as mean ± SD and tests for differences in the means are used in one-way analysis of variance and unpaired, 2-tailed *t* tests. Continuous variables without a normal distribution are presented as median (IQR) and were compared using the Wilcoxon rank-sum test. Categorical variables were compared using chi-squared tests.

Incidence rates per 100,000 person-years were calculated, and incidence rate ratios were assessed using Poisson’s regression. Incidence rates and ratios were restricted to those without prevalent AF, defined as the presence of AF before or on the recruitment date (n = 2,226).

Unadjusted and adjusted Cox proportional hazards regression models were used to assess the relationship between ethnicity and incident AF. Regression analyses were restricted to those without prevalent AF. Participants were censored at the time of their AF diagnosis, death, or the end of the study period (June 30, 2020), whichever came first.

Comparisons of a combined endpoint, including ischemic stroke and systemic infarct incidence, in those with AF according to ethnicity were made using Cox proportional hazards regression models before and after multivariable adjustment. Given a small population of Black, South Asian, Chinese, and multiracial individuals with AF and in order to obtain as full an assessment as possible regarding stroke and systemic infarcts as a complication of AF, all participants with prevalent AF (thus, any history of AF prior to and including the recruitment date) in addition to anyone with any AF throughout follow-up were included in the stroke and systemic infarct analyses. Participants with prevalent ischemic stroke or systemic infarct were excluded from the analyses (n = 9,685). For incident ischemic stroke and systemic infarcts, participants were censored at the time of their first ischemic stroke or systemic infarct diagnosis after the baseline visit, at death, or at the end of the study period.

A sensitivity analysis was conducted that limited the outcome to stroke alone. Participants with prevalent stroke were excluded (n = 5,718). Interaction analyses were performed to determine whether ethnicity was an effect modifier of the AF-stroke relationship.

Potential confounders and mediators were included as covariates in multivariable models based on previous literature. This included age, sex, education, body mass index, diabetes, hypertension, coronary disease, valvular disease, congestive heart failure, chronic kidney disease, obstructive sleep apnea, smoking, and alcohol.

A 2-tailed *P* < 0.05 was considered to be statistically significant. All statistical analyses were performed using SAS (version 9.4).

## Results

### Baseline characteristics

A total of 458,785 participants (438,333 White, 7,244 Black, 9,143 South Asian, 1,376 Chinese, and 2,689 multiracial) were included. The baseline characteristics between groups were heterogeneous and exhibited statistically significant differences in every covariate assessed except for valvular disease (Table 1). White individuals were, on average, older, less educated, more often had prevalent AF, and consumed alcohol more frequently.

**Table 1:** Baseline Characteristics

	White (n = 438,333)	Black (n = 7,244)	South Asian (n = 9,143)	Chinese (n = 1,376)	Multiracial (n = 2,689)	*P* Value
Age, y	57 ± 8	52.2 ± 8.2	53.6 ± 8.5	52.7 ± 7.7	52 ± 8.2	<0.001
Female	239,828 (54.7)	4,302 (59.4)	4,288 (46.9)	880 (64)	1,791 (63.3)	<0.001
Education						<0.001
O levels or equivalent	141,997 (33)	1,759 (25.2)	2,194 (25.6)	316 (24.3)	776 (29.6)	
CSEs or equivalent	52,489 (12.2)	1,335 (19.2)	949 (11)	83 (6.4)	386 (14.7)	
College or university degree	135,800 (31.5)	2,307 (33.1)	3,460 (40.3)	656 (50.4)	1,006 (38.4)	
Other professional	22,671 (5.3)	555 (8)	412 (4.8)	96 (7.4)	117 (4.5)	
None of the above	78,154 (18)	1,012 (14.5)	1,564 (18.2)	150 (11.5)	338 (12.9)	
Mean body mass index, kg/m^2^	27.5 ± 4.8	29.6 ± 5.5	27.2 ± 4.4	24.2 ± 3.5	27.5 ± 5.3	<0.001
Prevalent atrial fibrillation	2,183 (0.5)	9 (0.1)	28 (0.3)	1 (0.07)	5 (0.2)	<0.001
Diabetes	14,068 (3.2)	456 (6.3)	1,123 (12.3)	43 (3.1)	92 (3.4)	<0.001
Hypertension	62,259 (14.2)	1,336 (18.4)	1,811 (19.8)	125 (9.1)	301 (11.2)	<0.001
Coronary disease	15,254 (3.5)	142 (2)	620 (6.8)	22 (1.6)	55 (2)	<0.001
Valvular disease	4,734 (1.1)	74 (1)	87 (1)	11 (0.8)	18 (0.7)	0.15
Congestive heart failure	17,141 (3.9)	238 (3.3)	528 (5.8)	37 (2.7)	102 (3.8)	<0.001
Chronic kidney disease	26,901 (6.1)	513 (7.1)	932 (10.2)	41 (3)	117 (4.4)	<0.001
Obstructive sleep apnea	3,217 (0.7)	59 (0.8)	85 (0.9)	3 (0.2)	20 (0.7)	0.035
Smoking						<0.001
Prefer not to answer	1,606 (0.4)	55 (0,8)	106 (1.2)	4 (0.3)	14 (0.5)	
Never	233,150 (53.2)	5,003 (69.1)	6,992 (76.5)	1,085 (78.9)	1,273 (47.3)	
Previous	157,059 (35.8)	1,278 (17.6)	1,202 (13.1)	191 (13.9)	891 (33.1)	
Current	46,518 (10.6)	908 (12.5)	843 (9.2)	96 (7)	511 (19)	
Alcohol						<0.001
Prefer not to answer	328 (0.07)	28 (0.4)	56 (0.6)	2 (0.1)	5 (0.2)	
Never	30,425 (6.9)	1,616 (22.3)	4,175 (45.7)	385 (28)	314 (11.7)	
On special occasions	48,560 (11)	2,079 (28.7)	1,663 (18.2)	486 (35.3)	496 (18.4)	
1-3 times a month	49,230 (11.2)	942 (13)	663 (7.3)	150 (10.9)	390 (14.5)	
1-2 times a week	115,238 (26.3)	1,429 (19.7)	1,220 (13.3)	180 (13)	636 (23.7)	
3-4 times a week	103,190 (23.5)	692 (9.6)	754 (8.2)	81 (5.9)	450 (16.7)	
Daily	91,362 (21)	458 (6.3)	612 (6.7)	92 (6.7)	398 (14.8)	

Values are mean ± SD, n (%), or median (IQR). Baseline characteristics of study population according to ethnicity.

CSE = Certificate of Secondary Education; O levels = ordinary levels.

### Ethnicity and atrial fibrillation

**Figure 1:**
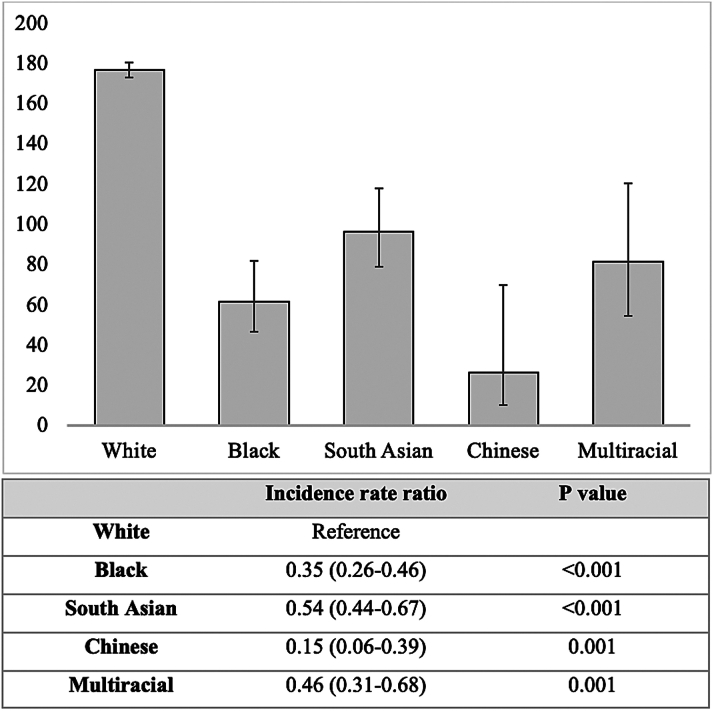
**Atrial Fibrillation Incidence Rates Per 100,000 Person-Years and Incidence Rate Ratios According to Ethnicity** Incidence rates per 100,000 person-years (y-axis) of atrial fibrillation according to ethnicity (x-axis). Error bars denote 95% CIs. Columns below show incidence rate ratios with White ethnicity used as reference.

During a mean follow-up of 11.1 ± 1.7 years, 8,706 participants exhibited incident AF. The type of AF first detected according to ethnicity is presented in Supplemental Table 2. Incidence rates of AF were significantly lower in all underrepresented ethnic groups compared to White individuals (Figure 1).

**Figure 2:**
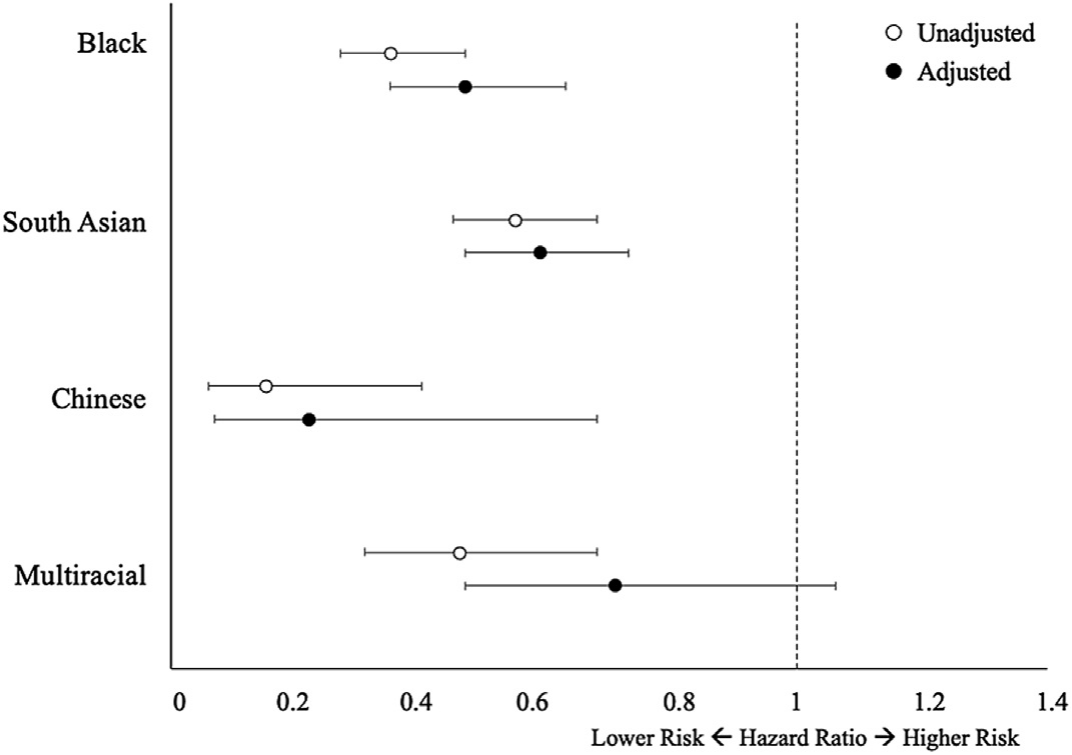
**Risk of Incident Atrial Fibrillation According to Ethnicity** Risk of incident atrial fibrillation according to ethnicity in unadjusted (open circles) and adjusted (filled circles) models. White ethnicity was used as the reference. Number of events = 8,706. Analyses were adjusted for age, sex, education, body mass index, diabetes, hypertension, coronary disease, valvular disease, congestive heart failure, chronic kidney disease, obstructive sleep apnea, smoking, and alcohol. Error bars denote 95% CIs.

Black, South Asian, Chinese, and multiracial individuals each exhibited substantially lower risks of incident AF compared to White individuals in unadjusted analyses, which remained significant for Black, South Asian, and Chinese individuals after multivariable adjustment (Figure 2, Central Illustration).

**Central Illustration:**
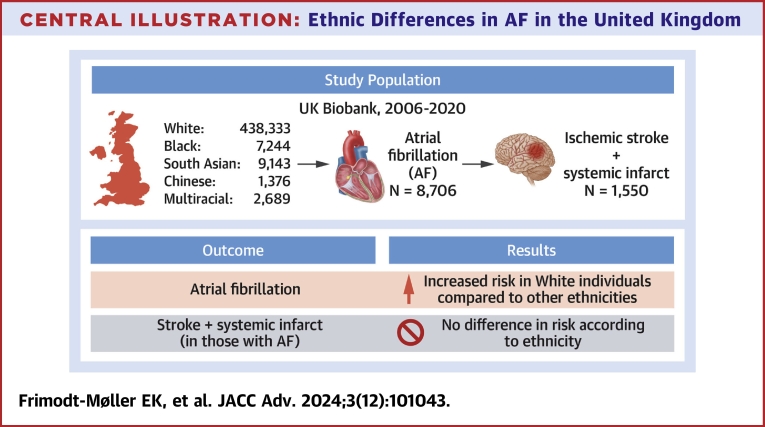
**Ethnic Differences in AF in the United Kingdom** Longitudinal data from the UK Biobank was leveraged to investigate whether ethnicity was associated with the risk of incident AF, AF-related stroke and systemic infarcts. Relative to other ethnic groups, White individuals exhibited a substantially higher risk of AF independent of established risk factors. No detectable differences in AF-related stroke and systemic infarct risk between ethnicities were observed. Analyses were adjusted for age, sex, education, body mass index, diabetes, hypertension, coronary disease, congestive heart failure, valvular disease, chronic kidney disease, obstructive sleep apnea, smoking, and alcohol consumption. AF = atrial fibrillation

### Atrial fibrillation-related stroke and systemic infarcts

Among those without prevalent stroke or systemic infarcts, AF was detected in 10,172 (prior to recruitment or during follow-up). Incident ischemic stroke and systemic infarcts occurred in 1,550 (1,513 in White individuals vs 37 in the rest of the cohort with AF). The number of events according to each ethnic subgroup is presented in Supplemental Table 3. There were no differences in the risk of ischemic stroke and systemic infarcts among those of the underrepresented ethnic populations compared to White individuals nor were there any differences in risk when stratified by each ethnicity (Table 2, Supplemental Figure 1). In sensitivity analyses, multiracial individuals had a higher risk of ischemic stroke in both unadjusted (HR: 2.42; 95% CI: 1.15-5.10; *P* = 0.02) and adjusted analyses (HR: 2.25; 95% CI: 1.01-5.04; *P* = 0.048) compared to White individuals, whereas there were no differences in the risk of stroke between the remaining ethnicities relative to White individuals (Table 3). However, small numbers of ischemic stroke and systemic infarct events among the underrepresented ethnic groups were present. Ethnicity did not modify the relationship between AF and ischemic stroke (*P* = 0.71).

**Table 2:** Risk of Ischemic Stroke or Systemic Infarct According to Ethnicity in Those With Atrial Fibrillation

	Unadjusted		Adjusted	
	HR (95% CI)	*P* Value	HR (95% CI)	*P* Value
Black	1.26 (0.68-2.36)	0.46	1.51 (0.80-2.82)	0.19
South Asian	1.09 (0.68-1.73)	0.73	0.81 (0.47-1.41)	0.46
Chinese	1.28 (0.18-9.06)	0.81	N/A^a^	N/A^a^
Multiracial	2.21 (1.10-4.42)	0.03	2.04 (0.97-4.28)	0.06

White ethnicity was used as the reference. Number of events in White individuals = 1,513 of 9,973 with AF (15%), and in the rest of the cohort = 37 of 199 (19%). Analyses were adjusted for age, sex, education, body mass index, diabetes, hypertension, coronary disease, congestive heart failure, valvular disease, chronic kidney disease, obstructive sleep apnea, smoking, and alcohol consumption.

a Not applicable due to limited outcome data (number of events = 1).

**Table 3:** Risk of Ischemic Stroke According to Ethnicity in Those With Atrial Fibrillation

	Unadjusted		Adjusted	
	HR (95% CI)	*P* Value	HR (95% CI)	*P* Value
Black	1.28 (0.64-2.57)	0.49	1.49 (0.74-2.99)	0.27
South Asian	1.18 (0.71-1.97)	0.52	0.93 (0.51-1.70)	0.82
Chinese	1.74 (0.24-12.33)	0.58	N/A^a^	N/A^a^
Multiracial	2.42 (1.15-5.10)	0.02	2.25 (1.01-5.04)	0.048

Risk of ischemic stroke in each ethnicity in those with atrial fibrillation with White ethnicity as reference. Number of events in White individuals = 1,163 of 10,224 (11%) vs the rest of the cohort = 31 of 204 (15%). Adjusted models included age, sex, education, body mass index, diabetes, hypertension, coronary disease, valvular disease, congestive heart failure, chronic kidney disease, obstructive sleep apnea, smoking, and alcohol.

a Not applicable due to limited outcome data.

## Discussion

In the United Kingdom, White individuals experienced the highest risks of developing AF, consistent with multiple studies in the United States. Contrary to prior U.S. investigations, no statistically significant differences in the risk of ischemic stroke and systemic infarcts by ethnicity were observed, although insufficient power may have explained those negative results, and firm conclusions in relation to this should be drawn with caution.

To our knowledge, this is the first study investigating ethnic differences in AF and AF-related stroke and systemic infarct risk in a country outside the United States using longitudinal data from a large-scale population-based cohort. Previous studies outside the United States have been limited to: cross-sectional analyses,^19^^,^^20^ assessing individuals from both Europe, Asia, and North America utilizing different study design and ascertainment that varied by country,^21^ assessing hospitalized patients admitted with nonhemorrhagic stroke,^22^ or only involving patients with validated heart failure across Asia.^23^ Our study benefited from the size and quality of the UK Biobank, providing analyses from a comprehensive cohort including more than 450,000 individuals and allowing for linkage to longitudinal, national health data. While the majority of the study population consisted of White individuals, each of the underrepresented ethnic groups was still represented by thousands of individuals (each group representing larger numbers than those in many previous studies).^21^, ^22^, ^23^ However, the ethnic distribution may not be truly representative of the current UK population, where 81.7% of the population were considered ethnic White in the 2021 Census,^24^ limiting the generalizability of our findings. This highlights the need to improve recruitment of diverse ethnic populations in future studies to address such limitations and enhance generalizability across all ethnic groups.

### Addressing potential biases

A higher prevalence and incidence of AF among non-Hispanic White populations have been consistently observed.^4^, ^5^, ^6^^,^^10^^,^^25^ It is possible these observed differences occur as a result of racial or ethnic disparities due to differences related to symptom reporting, ascertainment of AF, or reduced health care access. Prior studies within the United States have attempted to address those potential explanations in several ways, including: performing negative control analyses (demonstrating different patterns among diagnoses other than AF),^4^ by showing more AF on baseline ECGs dictated by study cohort participation and therefore not relying on clinical health care settings,^5^ by assessing patients with continuous heart-rhythm monitoring via indwelling pacemakers,^26^ and by showing an increasing risk of AF among self-identified African Americans exhibiting more genotype-determined European ancestry.^10^ To our knowledge, only 1 study suggested that AF may be less common in non-Hispanic Whites. However, the possibility that non-Hispanic Whites more often had diagnosed AF treated with various rhythm control strategies (such as antiarrhythmic drugs and/or ablation) could not be excluded in that study.^27^

### Contributing factors to the ethnicity-atrial fibrillation relationship

Apart from issues of misclassification or ascertainment, other factors that may contribute to the elevated AF risk in White individuals are not well understood. These may include genetic effects,^10^^,^^11^ structural or hormonal effects such as larger left atrial diameter^5^ or higher atrial natriuretic peptide levels in White individuals,^7^ or differential environmental exposures related to ethnicity. In the current study population, White individuals were older and more likely to consume alcohol; both characteristics have been associated with higher risk of incident AF (both long-term alcohol consumption and as an acute trigger of a new AF episode).^28^, ^29^, ^30^ While these factors may have contributed to the increased risk of AF within White individuals in the present study, the observed ethnic differences persisted despite multivariable adjustment for both age and alcohol consumed.

Our findings in a country outside the United States that offers socialized medicine demonstrate that the phenomenon is not unique to the United States. As the United Kingdom is European and also predominately White, we cannot exclude the possibility that similar causal phenomena still play a role. However, as the geographic location and history of these countries are substantially different, the consistent findings now demonstrated outside the United States may provide additional evidence that AF risk is different as a function of ethnicity, regardless of the specific environment.

### Stroke and systemic infarcts risk

The major adverse consequence of AF is an ischemic stroke.^31^ In contrast to the consistent observations in the United States that under-represented racial and ethnic groups experience a higher risk of AF-related stroke,^13^^,^^32^^,^^33^ we were unable to demonstrate similarly statistically significant findings in the United Kingdom. The inclusion of systemic infarcts to increase the number of events (and therefore power) generally did not result in statistically significant results. Of note, sensitivity analyses did suggest a higher risk of ischemic stroke among multiracial individuals compared to White individuals; given the absence of similar findings in each ethnic group alone, this unique finding may reflect a cultural or social phenomenon rather than a biological one and is worthy of future study. While insufficient power to detect significant differences may explain our negative findings, those small numbers were driven by the fact that several ethnic groups exhibited a particularly low prevalence and incidence of AF. It is therefore possible that these observations represent a real phenomenon that is different than what has been observed in the United States. It is worth highlighting that White individuals in the United Kingdom were, on average, less educated than other groups. Although we adjusted for covariates found to be significantly different, these observations may yet demonstrate the importance of socioeconomic factors pertinent to AF-related stroke and systemic embolism.

### Study limitations

Important limitations must be acknowledged. This is an observational study describing epidemiologic phenomena, and the study is not designed to elucidate causal mechanisms. For example, differences in various therapies to prevent stroke, such as more guideline-adherent anticoagulation or control of hypertension with less disparities between ethnicities in the United Kingdom, may underlie the observations made. While associations observed may be explained by residual mediation or confounding, the identification of such responsible factors would in fact be valuable in revealing factors pertinent to AF risk. Our findings are generally dependent on the accuracy of self-reported ethnicity, but even though admixed populations exist, ethnicity self-identification has been demonstrated to correlate well with genetic ancestry^34^ and should capture social aspects of self-determined identity. As participants had to be interested and able (such as able to spare the time) to participate in the UK Biobank, a “healthy-volunteer” selection bias may be present—while this should not necessarily amplify differences between groups within the cohort, it may limit generalizability of our findings.^17^ Furthermore, we did not ascertain information on various therapies for AF, such as anti-arrhythmic drugs or anticoagulants, which may have influenced the presence of AF and AF-related stroke. We sought to mitigate this by identifying incident (or first-time) diagnoses of AF (where anti-arrhythmic drugs should be less relevant), but differences in subsequent receipt of stroke reduction therapies may be an explanatory factor in differences in AF-related stroke that were observed. Information on lifestyle behaviors such as alcohol consumption was based on ascertainment at baseline, and participants may have changed their behavior over time. However, prior research suggests most of the measured covariates are fairly stable within individuals over time.^35^, ^36^, ^37^ While socialized medicine at least theoretically should limit disparities in health care access and delivery, it is important to emphasize that other differences in exposures (including relative wealth, environmental factors, and other culturally-related behaviors) by ethnicity that are important to health and the outcomes studied here likely remain operative. While the current models adjusted for level of education, other social determinants of health may yet have influenced the differences observed. Finally, ascertainment of several comorbidities and outcomes relied on ICD-9 and ICD-10 codes, which may not be perfectly sensitive and wherein exact timing of a particular event can be difficult to accurately infer. However, examination of these codes vs chart review has revealed excellent sensitivity and specificity for AF,^38^ and research relying exclusively on these codes have yielded high-impact results validated by studies performed using conventional methods.^4^^,^^29^^,^^39^, ^40^, ^41^

## Conclusions

Independent of established AF risk factors, White individuals experienced a heightened risk of AF compared to other ethnic groups among research participants in the United Kingdom. In contrast to observations from U.S.-based data, no significant differences in AF-associated stroke or systemic embolism between ethnic groups were detected, although a relatively small number of events may have precluded sufficient power to detect differences. These findings from a population-based cohort outside the United States suggest that the ethnic differences in AF may be a global phenomenon not sufficiently explained by differences in health care access or environment. The absence of differences in stroke and systemic embolism among these AF patients in the United Kingdom may suggest extant disparities in these adverse outcomes in the United States are remediable.

PERSPECTIVES **COMPETENCY IN PATIENT CARE:** Our study contributes to the growing evidence that differential access to health care systems cannot entirely account for the observed differences in AF incidence by ethnicities or races. However, these data suggest that U.S.-based disparities in AF-related stroke and systemic infarcts need not apply in other national health care systems.

**TRANSLATIONAL OUTLOOK:** Understanding the etiologies of ethnic differences in AF incidence may uncover as yet unknown mechanisms of AF pertinent to prevention strategies or novel therapies. More research is warranted to examine whether ethnic differences in AF-related stroke, as seen in the United States, persist in other countries with socialized medicine. Identifying specific (and perhaps various) explanations underlying the AF-associated stroke and systemic infarct risk differences may reveal useful strategies to assure that AF patients can achieve equity in the United States in regard to potential adverse outcomes of this common disease.

## Funding support and author disclosures

The UK Biobank has received funding from the 10.13039/501100000265UK Medical Research Council, 10.13039/100010269Wellcome Trust, Department of Health, 10.13039/501100000274British Heart Foundation, 10.13039/501100000361Diabetes UK, 10.13039/501100004186Northwest Regional Development Agency, 10.13039/100012095Scottish Government, and Welsh Assembly Government. As described in the manuscript, the MRC and Wellcome Trust played a key role in the decision to establish the UK Biobank, a large, population-based, prospective, open-access resource that would allow detailed investigations of the genetic and environmental determinants of the diseases of middle and old age. The MRC, Wellcome Trust, Department of Health, and Scottish Chief Scientist Office each have a representative on the UK Biobank Board. The MRC and Wellcome Trust fund the independent Ethics and Governance Council.^16^ Dr Biering-Sørensen is the chief investigator of the Novo Nordisk financed “NUDGE-DM-PERSISTENCE” trial; chief investigator of the Boston Scientific financed “DANLOGIC-HF” trial; chief investigator of the Sanofi Pasteur financed “NUDGE-FLU” trial; chief investigator of the Sanofi Pasteur financed “DANFLU-1” trial; chief investigator of the Sanofi Pasteur financed “DANFLU-2” trial; steering committee member of Boston Scientific sponsored “LUX-Dx TRENDS Evaluates Diagnostics Sensors in Heart Failure Patients Receiving Boston Scientific's Investigational ICM System” trial; steering committee member of the Amgen sponsored GALACTIC-HF trial; steering committee member of the Boehringer Ingelheim financed EASi-KIDNEY trial; advisory board member of Sanofi Pasteur, Amgen, CSL Seqirus, and GSK; has received speaker honorarium from Bayer, Novartis, Sanofi Pasteur, GE Healthcare, and GSK; has received research grants from Boston Scientific, GE Healthcare, AstraZeneca, Novo Nordisk, and Sanofi Pasteur; and has consultant appointments with Novo Nordisk, IQVIA, and Parexel. Dr Jackson has received research grants from the National Institute of Health, specifically the National Institute on Minority Health and Health Disparities, is currently supported by 1K01HL159041 from the National Heart, Lung and Blood Institute and the American Association under award number 851386; serves as a consultant to Biosense Webster Inc, Johnson & Johnson, Sanofi, Bristol Myers Squibb, and Pfizer; and receives honoraria from Zoll LifeVest, CME Outfitters, Health Monitor, PRIME Education, and WebMD/Medscape. Dr Marcus has received funding from the NIH (NHLBI, NIBIB, and NCI), TRDRP, and PCORI; and is a consultant for and has equity in InCarda. All other authors have reported that they have no relationships relevant to the contents of this paper to disclose.
