# Supplementary material for: Ethnic Differences in Atrial Fibrillation in the United Kingdom
Source: JACC Adv. 2024 Jul 3;3(12):101043. doi: 10.1016/j.jacadv.2024.101043 (PMC11734043; doi:10.1016/j.jacadv.2024.101043)
Supplement: Supplemental Figures and Tables [file mmc1.docx]

**Supplemental Table 1. Outcome and Baseline Comorbidities Assessment from International classification of Diseases, Ninth Revision (ICD-9) and International Statistical Classification of Diseases and Related Health Problems, Tenth Revision (ICD-10) codes**

|  | **ICD-9** | **ICD-10** |
| --- | --- | --- |
| **Atrial fibrillation** | 427.31 | I48.0, I48.1, I48.11, I48.19, I48.2, I48.20, I48.21  I48.91 |
| **Non-hemorrhagic stroke or transient ischemic attack** | 433.01, 433.11, 433.21, 433.31, 433.81, 433.91, 434, 434.0, 434.01, 434.1, 434.10, 434.11, 434.9, 434.91, 435, 435.8, 435.9 | I63, I63.0, I63.00, I63.01, I63.011, I63.012, I63.013, I63.019. I63.02. I63.03, I63.031, I63.032, I63.033, I63.039, I63.09, I63.1, I63.10, I63.11, I63.111, I63.112,  I63.113, I63.119, I63.12, I63.13, I63.131, I63.132,  I63.133, I63.139, I63.19, I63.2, I63.20, I63.21, I63.211,  I63.212, I63.213, I63.219, I63.22, I63.23, I63.231, I63.232, I63.233, I63.239, I63.29, I63.3, I63.30, I63.31,  I63.311, I63.312, I63.313, I63.319, I63.32, I63.321, I63.322, I63.323, I63.329,  I63.33, I63.331, I63.332,  I63.333, I63.339, I63.34, I63.341, I63.342, I63.343, I63.349, I63.39, I63.4, I63.40  I63.41, I63.411, I63.412, I63.413, I63.419, I63.42, I63.421, I63.422, I63.423,  I63.429, I63.43, I63.431,  I63.432, I63.433, I63.439,  I63.44, I63.441, I63.442, I63.443, I63.449, I63.5, I63.50, I63.51, I63.511, I63.512, I63.513, I63.519, I63.52, I63.521, I63.522, I63.523, I63.529, I63.53, I63.531, I63.532, I63.533, I63.539, I63.54, I63.541, I63.542, I63.543, I63.549, I63.59, I63.6  I63.8, I63.81, I63.89, I63.9, I67.82, G45, G45.0, G45.1  G45.2, G45.3, G45.4, G45.8, G45.9 |
| **Systemic infarctions** | | |
| **Retinal infarction** | 362.3, 362.30, 362.31, 362.32, 362.33, 362.34, 362.84, 388.02 | H34, H34.01, H34.02, H34.03, H34.10, H34.11, H34.12, H34.13, H34.211, H34.212, H34.213, H34.219, H34.231, H34.232, H34.233, H34.239 |
| **Ear infarction** | 388.01 | H93.01, H93.011, H93.012, H93.013, H93.019 |
| **Pulmonary embolism** | 415.1, 415.13, 415.19, 416.2 | I26, I26.0, I26.02, I26.09, I26.9, I26.92, I26.93, I26.94, I26.99, I27.82, Z86.711 |
| **Thoric aorta embolism** | 444.1 | I74.11 |
| **Abdominal aorta embolism** | 444.0, 444.01, 444.09 | I74.0, I74.01, I74.09 |
| **Mesenteric infarction** | 557.0 | K55.0, K55.01, K55.011, K55.012, K55.019, K55.02, K55.021, K55.022, K55.029, K55.03, K55.031, K55.032, K55.039, K55.04, K55.041, K55.042, K55.049, K55.05, K55.051, K55.052, K55.059, K55.06, K55.061, K55.062, K55.069 |
| **Hepatic infarction** | 573.4 | K76.3 |
| **Renal infarction** | 445.81, 593.81 | N28.0 |
| **Extremities** | 440.4, 444.2, 444.21, 445.01, 444.22, 445.02, 444.81 | I74.4, I75.0, I74.2, I75.01, I75.011, I75.012, I75.013, I75.019, I74.3, I74.5, I75.02, I75.021, I75.022, I75.023, I75.029 |
| **Other infarctions** | 444.8, 444.89, 444.9, 445.89 | I74.1, I74.10, I74.19, I74.8, I74.9, I75.89 |
| **Comorbidities** |  |  |
| **Diabetes** | 250, 250.0, 250.00, 250.01, 250.02, 250.03, 250.1, 250.10, 250.11, 250.12, 250.13, 250.2, 250.20, 250.21, 250.22, 250.23, 250.3, 250.30, 250.31, 250.32, 250.33, 250.4, 250.40, 250.41, 250.42, 250.43, 250.5, 250.50, 250.51, 250.52, 250.53, 250.6, 250.60, 250.61, 250.62, 250.63, 250.7, 250.70, 250.71, 250.72, 250.73, 250.8, 250.80, 250.81, 250.82, 250.83, 250.9, 250.90, 250.91, 250.92, 250.93 | E10, E10.1, E10.10, E10.11, E10.2, E10.21, E10.22, E10.29, E10.3, E10.31, E10.311, E10.319, E10.32, E10.321, E10.3211, E10.3212, E10.3213, E10.3219, E10.329, E10.3291, E10.3292, E10.293, E10.3299, E10.33, E10.331, E10.3311, E10.3312, E10.3313, E10.3319, E10.339, E10.3391, E10.3392, E10.3393, E10.3399, E10.34, E10.341, E10.3411, E10.3412, E10.3413, E10.3410, E10.349, E10.3491, E10.3492, E10.3493, E10.3499, E10.35, E10.351, E10.3511, E10.3512, E10.3513, E10.3519, E10.35, E10.3521, E10.3522, E10.3523, E10.3529, E10.353, E10.3531, E10.3532, E10.3533, E10.3539, E10.354, E10.3541, E10.3542, E10.3543, E10.3549, E10.355, E10.3551, E10.3552, E10.3553, E10.3559, E10.359, E10.3591, E10.3592, E10.3593, E10.3599, E10.36, E10.37, E10.37x1, E10.37x2, E10.37x3, E10.37x9, E10.39, E10.4, E10.40, E10.41, E10.42, E10.43, E10.44, E10.49, E10.5, E10.51, E10.52, E10.59, E10.6, E10.61, E10.610, E10.618, E10.62, E10.620, E10.621, E10.622, E10.628, E10.63, E10.630, E10.638, E10.64, E10.641, E10.649, E10.65, E10.69, E10.8, E10.9, E11, E11.0, E11.00, E11.01, E11.1, E11.10, E11.11, E11.2, E11.21, E11.22, E11.29, E11.3, E11.31, E11.311, E11.319, E11.32, E11.321, E11.3211, E11.3212, E11.3213, E11.3219, E11.329, E11.3291, E11.3292, E11.3293, E11.3299, E11.33, E11.331, E11.3311, E11.3312, E11.3313, E11.3319, E11.339, E11.3391, E11.3392, E11.3393, E11.3399, E11.34, E11.341, E11.3411, E11.3412, E11.3413, E11.3419, E11.349, E11.3491, E11.3492, E11.3493, E11.3499, E11.35, E11.351, E11.3511, E11.3512, E11.3513, E11.3519, E11.352, E11.3521, E11.3522, E11.3523, E11.3529, E11.353, E11.3531, E11.3532, E11.3544, E11.3539, E11.354, E11.3541, E11.3542, E11.3543, E11.3549, E11.355, E11.3551, E11.3552, E11.3553, E11.3559, E11.359, E11.3591, E11.3592, E11.3953, E11.3599, E11.36, E11.37, E11.37x1, E11.37x2, E11.37x3, E11.37x9, E11.39, E11.4, E11.40, E11.41, E11.42, E11.43, E11.44, E11.49, E11.5, E11.51, E11.52, E11.59, E11.6, E11.61, E11.610, E11.618, E11.62, E11.620, E11.621, E11.622, E11.628, E11.63, E11.630, E11.638, E11.64, E11.641, E11.649, E11.65, E11.69, E11.8, E11.9, E13, E13.0, E13.00, E13.01, E13.1, E13.10, E13.11, E13.2, E13.21, E13.22, E13.29, E13.3, E13.31, E13.311, E13.319, E13.32, E13.321, E13.3211, E13.3212, E13.3213, E13.3219, E13.329, E13.3291, E13.3292, E13.3293, E13.3299, E13.33, E13.331, E13.3311, E13.3312, E13.3313, E13.3319, E13.339, E13.3391, E13.3392, E13.3393, E13.3399, E13.34, E13.341, E13.3411, E13.3412, E13.3413, E13.3419, E13.349, E13.3491, E13.3492, E13.3493, E13.3499, E13.35, E13.351, E13.3511, E13.3512, E13.3513, E13.3519, E13.352, E13.3521, E13.3522, E13.3523, E13.3529, E13.353, E13.3531, E13.3532, E13.3533, E13.3539, E13.354, E13.3541, E13.3542, E13.3543, E13.3549, E13.355, E13.3551, E13.3552, E13.3553, E13.3559, E13.359, E13.3591, E13.3592, E13.3593, E13.3599, E13.36, E13.37, E13.37x1, E13.37x2, E13.37x3, E13.37x9, E13.39, E13.4, E13.40, E13.41, E13.42, E13.43, E13.44, E13.49, E13.5, E13.51, E13.52, E13.59, E13.6, E13.61, E13.610, E13.618, E13.62, E13.620, E13.621, E13.622, E13.628, E13.63, E13.630, E13.638, E13.64, E13.641, E13.649, E13.65, E13.69, E13.8, E13.9 |
| **Hypertension** | 401, 401.0, 401.1, 401.9, 402, 402.0, 402.01, 402.1, 402.10, 402.11, 402.9, 402.90, 402.91, 403, 403.0, 403.00, 403.01, 403.1, 403.10, 403.11, 403.9, 403.90, 403.91, 404, 404.0, 404.00, 404.01, 404.02, 404.03, 404.1, 404.10, 404.11, 404.12, 404.13, 404.9, 404.90, 404.91, 404.92, 404.93, 405, 405.0, 405.01, 405.09, 405.1, 405.11, 405.19, 405.9, 405.91, 405.99, 437.2 | I10, I11, I11.0, I11.9, I12, I12.0, I12.9, I13, I13.0, I13.1, I13.10, I13.11, I13.2, I15, I15.0, I15.1, I15.2, I15.8, I15.9 |
| **Coronary disease** | 414.0, 414.00, 414.01, 414.02, 414.03, 414.04, 414.05, 414.06, 414.07 | I25, I25.1, I25.10, I25.11, I25.110, I25.111, I25.112, I25.118, I25.119, I25.7, I25.70, I25.700, I25.701, I25.702, I25.708, I25.709, I25.71, I25.710, I25.711, I25.712, I25.718, I25.719, I25.72, I25.720, I25.721, I25.722, I25.728, I25.729, I25.73, I25.730, I25.731, I25.732, I25.738, I25.739, I25.75, I25.750, I25.751, I25.752, I25.758, I25.759, I25.76, I25.760, I25.761, I25.762, I25.768, I25.769, I25.79, I25.790, I25.791, I25.792, I25.798, I25.799, I25.81, I25.810, I25.811, I25.812, I25.82, I25.83, I25.84, I25.89, I25.9 |
| **Valvular disease** | 396, 396.0, 396.1, 396.2, 396.3, 396.8, 396.9 | I05, I05.0, I05.1, I05.2, I05.8, I05.9, I06, I06.0, I06.1, I06.2, I06.8, I06.9, I07, I07.0, I07.1, I07.2, I07.8, I07.9, I08, I08.0, I08.1, I08.2, I08.3, I08.8, I08.9, I09, I09.0, I09.1, I09.2, I09.8, I09.81, I09.89, I09.9, I34, I34.0, I34.1, I34.2, I34.8, I34.81, I34.89, I34.9, I35, I35.0, I35.1, I35.2, I35.8, I35.9, I36, I36.0, I36.1, I36.2, I36.8, I36.9, I37, I37.0, I37.1, I37.2, I37.8, I37.9 |
| **Congestive heart failure** | 428, 428.0, 428.1, 428.2, 428.20, 428.21, 428.22, 428.23, 428.3, 428.30, 428.31, 428.32, 428.33, 428.4, 428.40, 428.41, 428.42, 428.43, 428.9 | I11.0, I13.0, I13.2, I50, I50.1, I50.2, I50.20, I50.21, I50.22, I50.23, I50.3, I50.30, I50.31, I50.32, I50.33, I50.4, I50.40, I50.41, I50.42, I50.43, I50.8, I50.81, I50.810, I50.811, I50.812, I50.813, I50.814, I50.82, I50.83, I50.84, I50.89, I50.9 |
| **Chronic kidney disease** | 585, 585.1, 585.2, 585.3, 585.4, 585.5, 585.6, 585.9, 586, 587 | I12, I12.0, I12.9, I13, I13.0, I13.1, I13.10, I13.11, I13.2, N18, N18.1, N18.2, N18.3, N18.30, N18.31, N18.32, N18.4, N18.5, N18.6, N18.9, N19 |
| **Obstructive sleep apnea** | 327.23 | G47.3, G47.30, G47.31, G47.32, G47.33, G47.34, G47.35, G47.36, G47.37, G47.39 |

**Supplemental Table 2. Type of Atrial Fibrillation First Detected During Study Period According to Ethnicity**

| **Type of atrial fibrillation** | **White**  **n=438,333** | **Black**  **n=7,244** | **South Asian**  **n=9,143** | **Chinese**  **n=1,376** | **Multiracial**  **n=2,689** | **Total** |
| --- | --- | --- | --- | --- | --- | --- |
| **Paroxysmal** | 5,174 (1.2%) | 29  (0.4%) | 59  (0.6%) | 3  (0.2%) | 15  (0.6%) | 5,280 |
| **Persistent/Chronic** | 1,041  (0.2%) | 9  (0.1%) | 7  (0.1%) | 1  (0.1%) | 5  (0.2%) | 1,063 |
| **Paroxysmal & Persistent/Chronic same time** | 2,320  (0.5%) | 10  (0.1%) | 29  (0.3%) | 0 | 4  (0.2%) | 2,363 |
| **Total** | 8,535  (2%) | 48  (0.7%) | 95  (1%) | 4  (0.3%) | 24  (0.9%) | 8,706 |

**Supplemental Table 3.** **Number of Incident Ischemic Infarcts by Ethnicity among those with Atrial Fibrillation**

| **Ischemic Infarct during Follow-up** | **White** | **Black** | **South Asian** | **Chinese** | **Multiracial** |
| --- | --- | --- | --- | --- | --- |
| **No** | 8460  (85%) | 19  (70%) | 95  (84%) | 4  (80%) | 44  (81%) |
| **Yes** | 1513  (15%) | 8  (29%) | 18  (16%) | 1  (20%) | 10  (19%) |
| **Total** | 9973 | 27 | 113 | 5 | 54 |

**Supplemental Figure 1. Risk of Incident Ischemic Stroke or Systemic Infarct in those with Atrial Fibrillation in Minority Ethnic Groups compared to White Individuals**


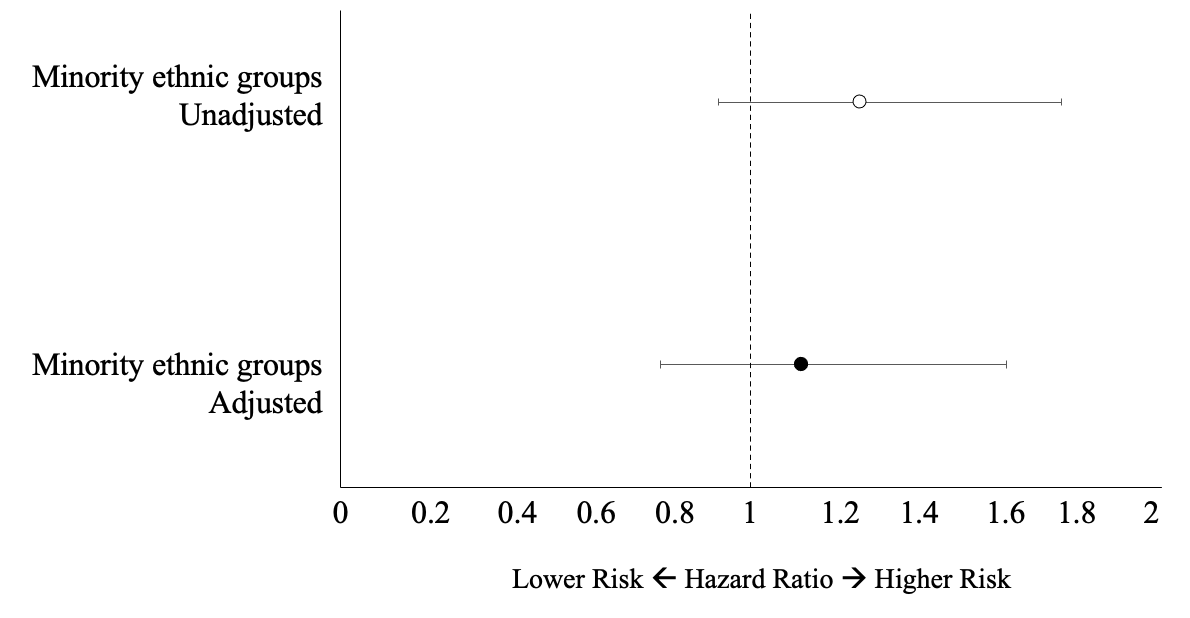


Risk of incident ischemic stroke or systemic infarct in minority ethnic groups in unadjusted (open circles) and adjusted (full circles) models. White ethnicity was used as the reference. Analyses were adjusted for age, sex, education, body mass index, diabetes, hypertension, coronary disease, valvular disease, congestive heart failure, chronic kidney disease, obstructive sleep apnea, smoking, and alcohol. Error bars denote 95% confidence intervals.
